# Inter-Trial Correlations in Predictive-Saccade Endpoints: Fractal Scaling Reflects Differential Control along Task-Relevant and Orthogonal Directions

**DOI:** 10.3389/fnhum.2017.00100

**Published:** 2017-03-07

**Authors:** Pamela Federighi, Aaron L. Wong, Mark Shelhamer

**Affiliations:** ^1^Department of Otolaryngology—Head and Neck Surgery, Johns Hopkins University School of MedicineBaltimore, MD, USA; ^2^University of FirenzeFirenze, Italy; ^3^Department of Neurology, Johns Hopkins University School of MedicineBaltimore, MD, USA

**Keywords:** motor control, fractal scaling, oculomotor

## Abstract

Saccades exhibit variation in performance from one trial to the next, even when paced at a constant rate by targets at two fixed locations. We previously showed that amplitude fluctuations in consecutive predictive saccades have fractal structure: the spectrum of the sequence of consecutive amplitudes has a power-law (*f* ^−α^) form, indicative of inter-trial correlations that reflect the storage of prior performance information to guide the planning of subsequent movements. More gradual decay of these inter-trial correlations coincides with a larger magnitude of spectral slope α, and indicates stronger information storage over longer times. We have previously demonstrated that larger decay exponents (α) are associated with faster adaptation in a saccadic double-step task. Here, we extend this line of investigation to predictive saccade endpoints (i.e., movement errors). Subjects made predictive, paced saccades between two fixed targets along a horizontal or vertical axis. Endpoint fluctuations both along (on-axis) and orthogonal to (off-axis) the direction of target motion were examined for correlations and fractal structure. Endpoints in the direction of target motion had little or no correlation or power-law scaling, suggesting that successive movements were uncorrelated (white noise). In the orthogonal direction, however, the sequence of endpoints did exhibit inter-trial correlations and scaling. In contrast, in our previous work the scaling of saccade amplitudes is strong along the target direction. This may reflect the fact that while saccade amplitudes are neurally programmed, endpoints are not directly controlled but instead serve as a source of error feedback. Hence, the lack of correlations in on-axis endpoint errors suggests that maximum information has been extracted from previous movement errors to plan subsequent movement amplitudes. In contrast, correlations in the off-axis component indicate that useful information still remains in this error (residual) sequence, suggesting that saccades are less tightly controlled along the orthogonal direction.

## Introduction

The saccadic system generates rapid eye movements that shift the fovea from one place to another in the visual scene (Leigh and Zee, 2006; Ramat et al., 2007). To ensure visual acuity, saccades must be fast and accurate (Becker, 1989; Kowler and Blaser, 1995). In most cases, saccades can be viewed as ballistic. In other words, once planned, a given saccade will be completed according to the programmed direction and amplitude, regardless of any intervening changes in the stimulus. Saccade accuracy is established by an efferent copy of the ongoing motor command (Robinson, 1975; Wolpert et al., 1995; Optican, 2005). This efferent copy is used to predict the sensory effects of the movement (Quaia et al., 2000), which are compared to the actual movement outcome. Information about movement errors is then stored and processed to adjust future saccade plans as needed to maintain accuracy.

The errors in saccadic eye movements have variable and systematic components (Aitsebaomo and Bedell, 1992; White et al., 1994). The systematic error reflects a consistent bias in the endpoints along the direction of target motion, generally resulting in hypometria (Kapoula and Robinson, 1986; Deubel, 1987; Collewijn et al., 1988a,b). The variable error represents inter-trial variability in the final saccade position and causes dispersion of end positions in space. This variable error reflects the inability to consecutively produce exactly identical saccades, and has been regarded in models as arising from signal-dependent noise in motor commands (Harris and Wolpert, 1998) and uncertainty in target localization (van Beers, 2007). Previous studies have found that the components of the systematic and variable errors along the direction of target motion (on-axis) and in the orthogonal direction (off-axis) have different magnitudes and may be unrelated (van Opstal and van Gisbergen, 1989; Barton and Sparks, 2001; Metzger et al., 2004). It has been proposed that these differences reflect an accumulation of random uncorrected noise across trials in directions that are not task relevant (van Beers et al., 2013), or alternatively an intentional effort to constrain variability to dimensions that do not impact overall success (Cohen and Sternad, 2009).

Of particular interest to the study of movement variability and planning of future movements is the generation of predictive saccades. Predictive saccades are movements made to expected locations of the visual target, and are encouraged by asking subjects to saccade between rapidly paced (~0.7 to 1.0 Hz) alternating visual stimuli. They are generated in anticipation of each target onset and are characterized by short latencies (on the order of −150–50 ms, too short for visual information about the upcoming target to be processed) (Shelhamer and Joiner, 2003). These short latencies imply that such movements are produced almost entirely based on previous performance errors (Shelhamer, 2005). Thus, the correlation structure of long sequences of predictive saccades reflects the storage and processing of previously observed errors that are used to plan future movements. We have previously found inter-trial correlations between the latencies (Shelhamer and Joiner, 2003) and amplitudes (Wong and Shelhamer, 2011) of consecutive predictive saccades, such that the series of consecutive latencies or amplitudes exhibits long memory across large numbers of saccades. The amplitudes of predictive saccades also exhibit evidence of trial-by-trial corrections in a direction-specific (right/left) manner (Wong and Shelhamer, 2011), suggesting that predictive-saccade amplitudes may be tightly regulated using performance information extracted from previous movements. Such strong inter-trial correlations, however, suggest that a large amount of information has to be stored about each trial, raising questions of biological feasibility and storage capacity.

One way for the brain to address the information-storage issue is to retain only error information that is task-relevant, such as errors that only lie along the primary direction of motion. Hence, the aim of this study was to explore the nature of error information that is used to maintain saccade accuracy in the on-axis and off-axis directions. By examining endpoint errors produced at the conclusion of the primary saccade (before any corrective saccades occur), we gain insight into differences in error processing along directions in which movement errors are highly task-relevant (on-axis) and less relevant (off-axis). This study provides insight into how the brain allocates computational resources for motor programming, and in particular how storage and processing of task-relevant information might be optimized.

## Methods

### Subjects

Nine healthy subjects (two males, seven females, age range 23–53 years) were tested. All subjects had no reported neurological or oculomotor problems. All participants gave written informed consent, as approved by the Western Institutional Review Board under contract with Johns Hopkins Medical Institutions. Seven subjects performed all four experimental tasks; two subjects were tested in the H condition only, as part of a previous study. Three subjects were not naïve to the purposes of the study.

### Eye movement recording apparatus

Eye movements were recorded with a scleral search coil (Skalar Medical BV, Delft, The Netherlands), while data was acquired on a PC-compatible computer running custom real-time experiment-control software, developed in-house. Horizontal and vertical positions of the left or right eye were recorded, sampled at 1,000 Hz and encoded in binary digital form with 12-bit resolution, corresponding to a system resolution of approximately 0.03° (Robinson, 1963).

Typically the recorded eye was chosen for the comfort and convenience of the subject, if he or she expressed a preference. The left eye was chosen by most subjects. Ocular dominance was not measured or controlled for. Given the highly conjugate nature of normal targeting saccades (otherwise there would be post-saccadic diplopia), it is unlikely that our findings are different between the two eyes. Furthermore, both eyes were viewing at all times, even though only the movements of one eye were recorded.

The visual stimulus for prompting eye movements was produced with a laser reflected via computer-controlled mirror onto a rear-projection screen, 100 cm from the subject's eyes. Two different visual targets were used: a red dot (diameter 0.1°) and a vertical line (thickness 0.1°, extent 5.4°). In order to minimize perturbations due to head movements, subjects sat in a stationary chair with their heads restrained by a bite bar. Sessions were conducted in darkness.

### Experiment design

We investigated the error corrections and processing used to maintain saccade accuracy during predictive saccades. We focused on endpoint errors to test for long-term correlations in time series consisting of sequences of errors made during all trials of each task. We therefore tested predictive saccades to horizontal, vertical, and oblique targets located eccentrically in fixed positions. Horizontal predictive saccades toward targets in the form of a vertical line were also investigated.

In task H (horizontal predictive saccades to point targets), the visual target jumped between two positions in the horizontal plane located at ±5° with respect to the vertical meridian.

In task V (vertical predictive saccades to point targets), the visual target jumped between two positions in the vertical plane located at ±5° with respect to the horizontal meridian.

In task O (oblique predictive saccades to point targets), the visual target jumped between two positions, 10° apart, in an oblique direction oriented +45° or −45° with respect to the orthogonal plane (randomly chosen for each participant). The targets were located symmetrically with respect to the center of the orthogonal plane.

Task H consisted of a sequence of 500 trials, whereas tasks V and O consisted of a sequence of 500 or 400 trials. To obtain predictive saccadic movements, the visual targets were presented at a pacing rate of 0.9 Hz for all tasks (Wong and Shelhamer, 2011). In all tasks with a point target, the subjects were instructed to make an eye movement to each target as it appeared.

In task LINE (horizontal predictive saccades to a vertical line), we adopted the same protocol as for horizontal predictive saccades, except that a vertical line was used as the target and moved between locations at ±5° with respect to the vertical meridian. Subjects were instructed to make saccades so as to look at the center of the vertical line each time it appeared.

Each task included a calibration block that immediately preceded the experimental block. Calibration recordings were obtained for 12 target positions in the horizontal, vertical, and oblique directions, spanning up to 5° in each direction. The targets were shown for 1,000 or 1,500 ms. Linear interpolation between calibration positions was used to provide a calibration factor for each recorded eye position.

### Data processing and analysis

Processing of recorded data was performed off-line by a semiautomatic algorithm. Digitized coil signals were low-pass filtered at 100 Hz. Eye velocity was calculated using an eight-point central-difference derivative algorithm (Inchingolo and Spanio, 1985; Federighi et al., 2011). Saccades were identified automatically based on a velocity threshold of 10°/s. The same threshold was used to detect the starting and ending times of saccades. The automatic selection of saccades was verified interactively.

We examined saccade accuracy for the first (primary) saccade generated in response to each target movement. Saccade accuracy was assessed by measuring endpoint error, defined as the angular distance between target position in degrees (*T*) and position of the eye at the conclusion of the primary saccade in degrees (*P*): |*P*| − |*T*| (assuming that eye and target are on the same side of midline so that they have the same sign). Undershoot is defined by a negative value and overshoot by a positive value. We determined two components of endpoint errors: along the direction of target motion (on-axis errors), and orthogonal to the direction of target motion (off-axis errors).

Saccadic endpoint error has variable and systematic components. We examined the variable component of the on-axis and off-axis endpoint errors of predictive saccades by analyzing the correlation structures of their time series. For each subject and condition, separate time series of on-axis and off-axis endpoint errors were created. Since saccade accuracy may differ for rightward vs. leftward or upward vs. downward saccades, we examined saccades in each direction of motion separately.

We analyzed the correlation structure of each time series composed of on-axis or off-axis endpoint errors. We looked for long-term correlations in our time series data by two approaches: a spectral-domain method and a time-domain method (Rangarajan and Ding, 2000).

A wide class of stationary stochastic processes with long-term correlations have autocorrelation functions that decay as a power-law function of time lag: *R*_x_(τ) ≈ τ^−β^, where 0 < β < 1. The power spectrum of this type of time series also decays as a power-law function of frequency: *S*_x_(*f*) ≈ *f* ^−α^. Here, *f* is frequency and τ is a time lag that quantifies delay of the time series x[n−τ] relative to its original version x[n] (Papoulis, 1984; Rangarajan and Ding, 2000). For a time series x[n], the power spectrum is the Fourier transform of its autocorrelation function. Thus, information on temporal correlations will be expressed in both the time and the frequency (spectral) domains. Since long-term correlations in the autocorrelation function are small and noisy, and are better reflected in the low-frequency band of the power spectrum, we assessed long-term correlations in the spectral domain. Specifically, we measured exponent α, the slope of a linear regression on the log-log plot of the power spectrum determined as the squared magnitude of the Fourier transform of the time series, to quantify long-term correlations.

Rescaled range analysis was then used to estimate self-similarity in the time domain, by evaluating fluctuations as a function of the duration of a time window (Bassingthwaighte et al., 1994; Beran, 1994; Taqqu et al., 1995). The rescaled range is the ratio of the range (span between minimum and maximum values, *R*) of partial sums of a time series in different time windows, to the standard deviation (*S*) within that window. For some types of processes with long-term correlations, the rescaled range decays as a power-law function of window duration (Δ*T*). This power law is characterized by the Hurst exponent *H: R/S* ≈ Δ*T*^*H*^. We determined the Hurst exponent by linear regression on the log-log plot of *R/S* vs. window duration (Δ*T*).

Finally, we checked the consistency of the results obtained by the two methods. The Hurst exponent for a signal with true long-term correlations is linearly related to the coefficient α of the power spectrum, described above. We verified the theoretical relationship between the Hurst exponent and exponent α: *H* = (1 + α)/2, in order to identify artifacts that could produce misleading results (Rangarajan and Ding, 2000).

Algorithms for processing and analysis were developed in Matlab (The MathWorks Inc., Natick, MA, USA). Statistical computations were performed using SPSS software (SPSS Inc., Chicago, IL).

## Results

### Variability is greater along the task-relevant direction

**Figure 1:**
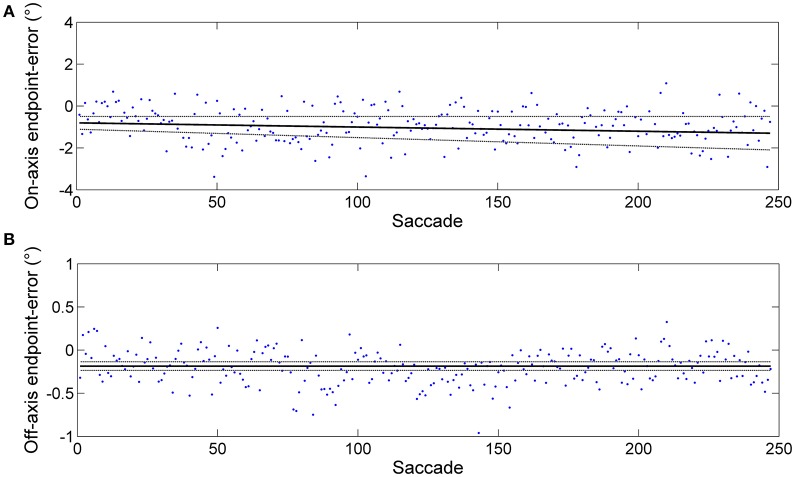
**Example of an endpoint-error time series obtained from horizontal predictive saccades made by a representative subject**. Data from rightward saccades are shown. The data were fitted using the linear polynomial equation: endpoint-error = *slope* × saccade + *const*. The 95% prediction bounds are indicated (broken curve). **(A)** Plot of endpoint-errors in the directions along target motion (on-axis) vs. trial number: slope = −0.002 ± 0.002 and const = −0.805 ± 0.303. **(B)** Plot of endpoint errors in the direction orthogonal to target motion (off-axis) vs. trial number: slope = −0.000 ± 0.000 and const = −0.186 ± 0.050. Despite generating a large number of predictive saccades (500, in this case), subject performance appears at least weakly stationary (constant mean and, to a close approximation, constant variance).

Endpoint-error time series for the point-target conditions were initially characterized with conventional summary statistics (Figure 1). Endpoint errors along the axis of target motion (on-axis errors) were larger than errors orthogonal to that direction (off-axis errors), for all conditions. On-axis errors (deg) were −0.62 ± 0.54 for Task H, −0.48 ± 0.30 for Task V, −0.56 ± 0.31 for Task O. Off-axis errors were 0.03 ± 0.30 for Task H, −0.03 ± 0.36 for Task V, −0.08 ± 0.21 for Task O. These differences were significant for all directions (*P* < 0.005). On-axis endpoint errors exhibited hypometria in the normal range as shown by others: an approximate 10% undershoot when target eccentricity is >10° (Becker and Fuchs, 1969; Henson, 1978; Kapoula and Robinson, 1986). The trend in the variable component of endpoint errors was estimated by measuring the sample variances. Significant differences in variances were found between on-axis and off-axis errors for all directions (*P* < 0.001). The variance was greater for on-axis errors [Task H: 1.35(0.72); Task V: 1.78(1.55); Task O: 1.20(0.60), median and interquartile range] than for off-axis errors [Task H: 0.07(0.04); Task V: 0.15(0.15); Task O: 0.34(0.05); median and interquartile range].

**Figure 2:**
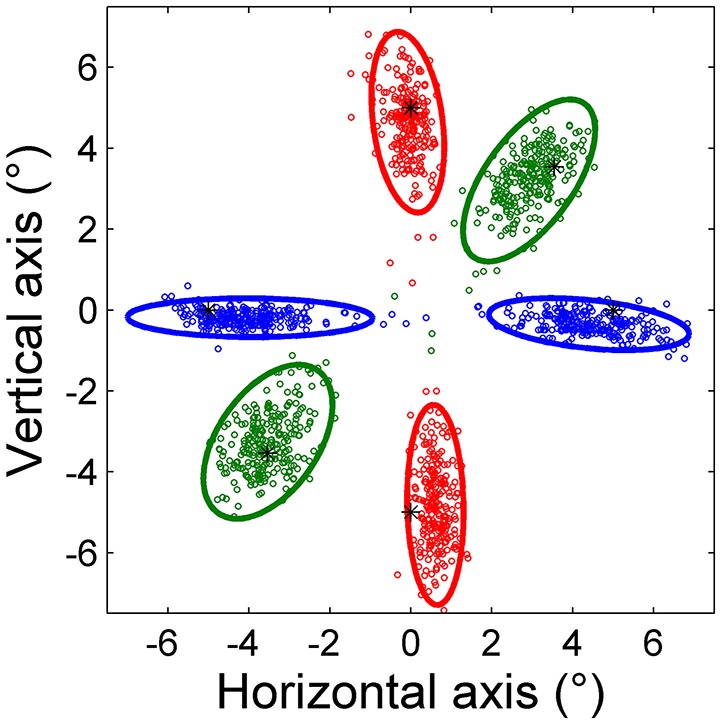
**Saccade endpoints of horizontal (blue), vertical (red), and oblique (green) predictive saccades made by a representative subject during Task H, Task V, and Task O, respectively**. All saccades were toward a point target, represented by an asterisk (^*^). The 95% confidence ellipses are shown.

Figure 2 shows saccade endpoints and endpoint variability. Variability is summarized by 95% confidence ellipses centered on the mean of the saccade endpoints. The data are from saccades performed by a representative subject in the three predictive tasks using point targets. The directions of the ellipse axes indicate that endpoint variability is approximately aligned with the direction of target movement, and the elongated shapes show that the variability of on-axis errors is greater than that of off-axis errors. Variability is more symmetric for oblique saccades, reflected in a more circular ellipse in these cases.

### Inter-trial correlations are weaker in the task-relevant direction

**Figure 3:**
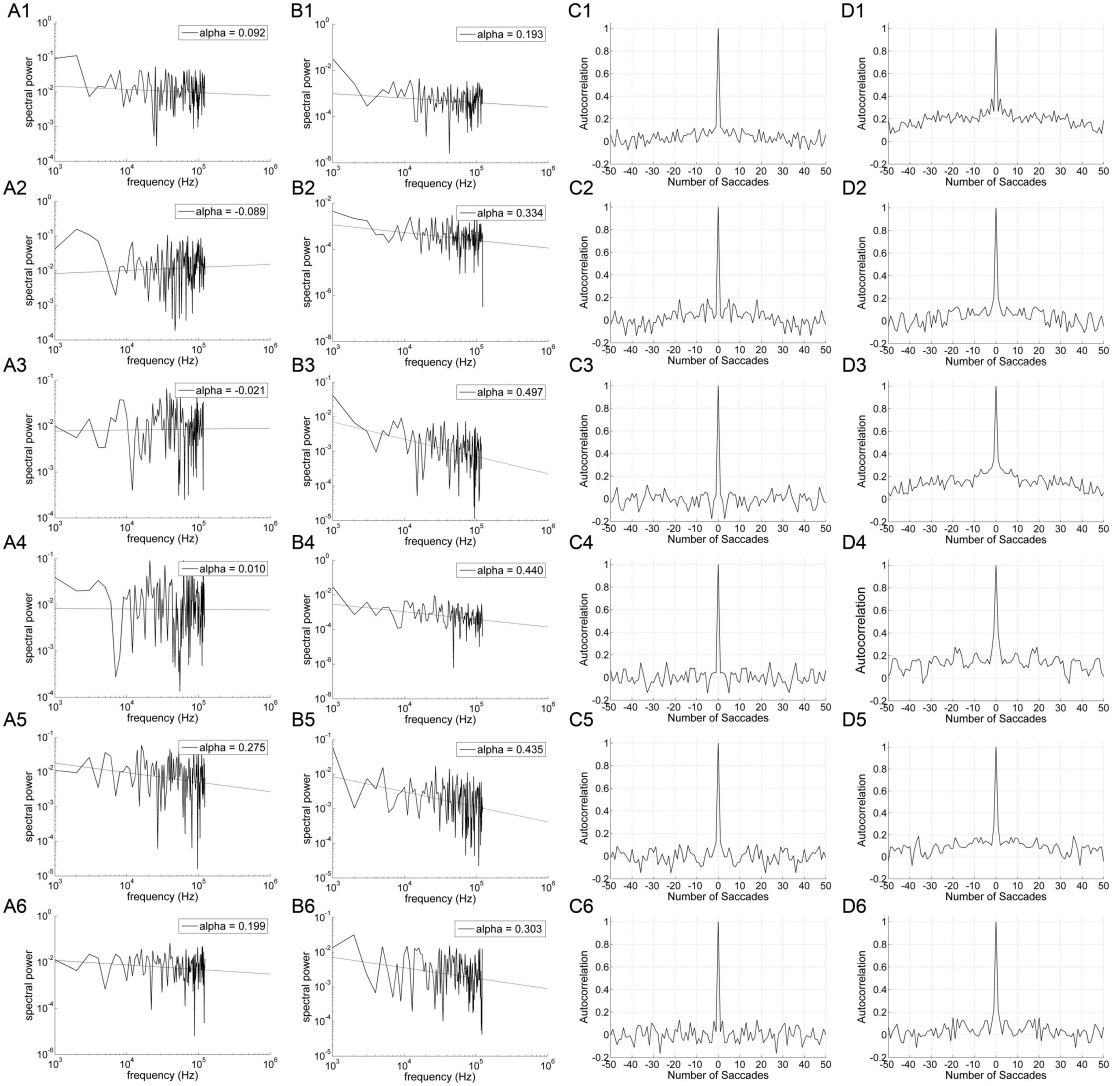
**An example of data obtained from 10° horizontal (rows A1–D1; A2–D2), vertical (rows A3–D3; A4–D4), and oblique (rows A5–D5; A6–D6) predictive saccades made by a representative subject**. Data from on-axis endpoint-error time series (columns **A** and **C**) are compared to data from off-axis endpoint-error time series (columns **B** and **D**). Data from rightward (1; 3; 5) and leftward (2; 4; 6) predictive saccades are shown separately. **(A,B)** Plot of power spectra of endpoint-error time series. Power spectra of data are plotted on a log-log scale. The broken line shows fit to a linear polynomial equation. Values of exponent α are indicated for the data represented. **(C,D)** Plot of corresponding autocorrelation functions of endpoint-error time series. Autocorrelation functions are plotted vs. the number of trials of relative shift between each endpoint-error time series and a copy of itself.

Figure 3 shows the power spectrum and autocorrelation function of the endpoint-error time series for a representative subject. The autocorrelation functions for on-axis endpoint-error time series of rightward and leftward saccade sequences decay more rapidly than for off-axis endpoint-error time series. The slow decay in autocorrelation functions indicates that off-axis endpoint-error time series exhibit correlations that persist longer than those for on-axis errors.

The power spectra provide a complementary means of visualizing these results, which better represents the longer-duration (lower-frequency) correlations in the error series. Data from horizontal and vertical predictive saccades generated by this subject show approximately flat power spectra for the on-axis direction, with values of α near zero that suggest white-noise processes and uncorrelated trials. On the contrary, the power spectra of off-axis endpoint-error time series show values of α in the range 0 < α < 1, indicating that the power spectrum decays as a power-law function of frequency. Power-law decay indicates correlations in off-axis endpoint errors of consecutive saccades. The pattern was similar for all subjects. Mean values of exponent α for rightward and leftward saccades are shown in Table 1.

**Table 1:** **Summary of scaling exponent α-values (mean and SD of α-values estimated for rightward and leftward saccades or upward and downward saccades in each experiment) from endpoint-error in the directions along target motion (on-axis) and orthogonal to target motion (off-axis), for all subjects**.

**Subject**	**Task H Target: dot**	**Task V Target: dot**	**Task O Target: dot**	**Task LINE Target: vertical line**
	**α on-axis/α off-axis**	**α on-axis/α off-axis**	**α on-axis/α off-axis**	**α on-axis/α off-axis**
1	0.00 ± 0.13/0.26 ± 0.10^*^	−0.01 ± 0.02/0.47 ± 0.04^*^	0.24 ± 0.05/0.37 ± 0.09^*^	0.05 ± 0.07/0.51 ± 0.06^*^
2	0.02 ± 0.07/0.16 ± 0.02^*^	0.08 ± 0.02/0.22 ± 0.06^*^	0.08 ± 0.02/0.33 ± 0.05^*^	0.25 ± 0.29/0.51 ± 0.02^*^
3	0.07 ± 0.07/0.55 ± 0.05^*^	0.43 ± 0.08/0.16 ± 0.06	0.48 ± 0.21/0.18 ± 0.21	−0.04 ± 0.02/0.72 ± 0.14^*^
4^a^	0.19 ± 0.33/0.36 ± 0.01^*^	−0.04 ± 0.07/0.16 ± 0.27^*^	0.21 ± 0.00/0.39 ± 0.22^*^	0.14 ± 0.19/0.73 ± 0.33^*^
5^a^	0.26 ± 0.04/0.52 ± 0.11^*^	0.18 ± 0.17/0.21 ± 0.00^*^	0.02 ± 0.27/0.20 ± 0.05^*^	0.32 ± 0.08/0.61 ± 0.29^*^
6	−0.01 ± 0.12/0.42 ± 0.30^*^	0.30 ± 0.14/0.48 ± 0.17^*^	0.34 ± 0.06/0.31 ± 0.05	0.14 ± 0.09/0.55 ± 0.09^*^
7	0.30 ± 0.16/0.20 ± 0.01	0.09 ± 0.29/0.63 ± 0.00^*^	0.59 ± 0.02/0.43 ± 0.10	0.41 ± 0.18/0.86 ± 0.12^*^
8	−0.08 ± 0.15/0.62 ± 0.32^*^			
9	0.16 ± 0.19/0.49 ± 0.05^*^			

Asterisks (^*^) indicate values of exponent α that were greater for off-axis errors than for on-axis errors. Each value in the table is the mean of the two exponents α calculated separately for either rightward and leftward saccades or for upward and downward saccades.

a *subject who skipped a saccade to one or more target jumps*.

Data obtained from oblique predictive saccades do not show a consistent trend in on-axis vs. off-axis endpoint-error time series (see Table 1).

### Scaling exponents are smaller along the task-relevant direction

**Figure 4:**
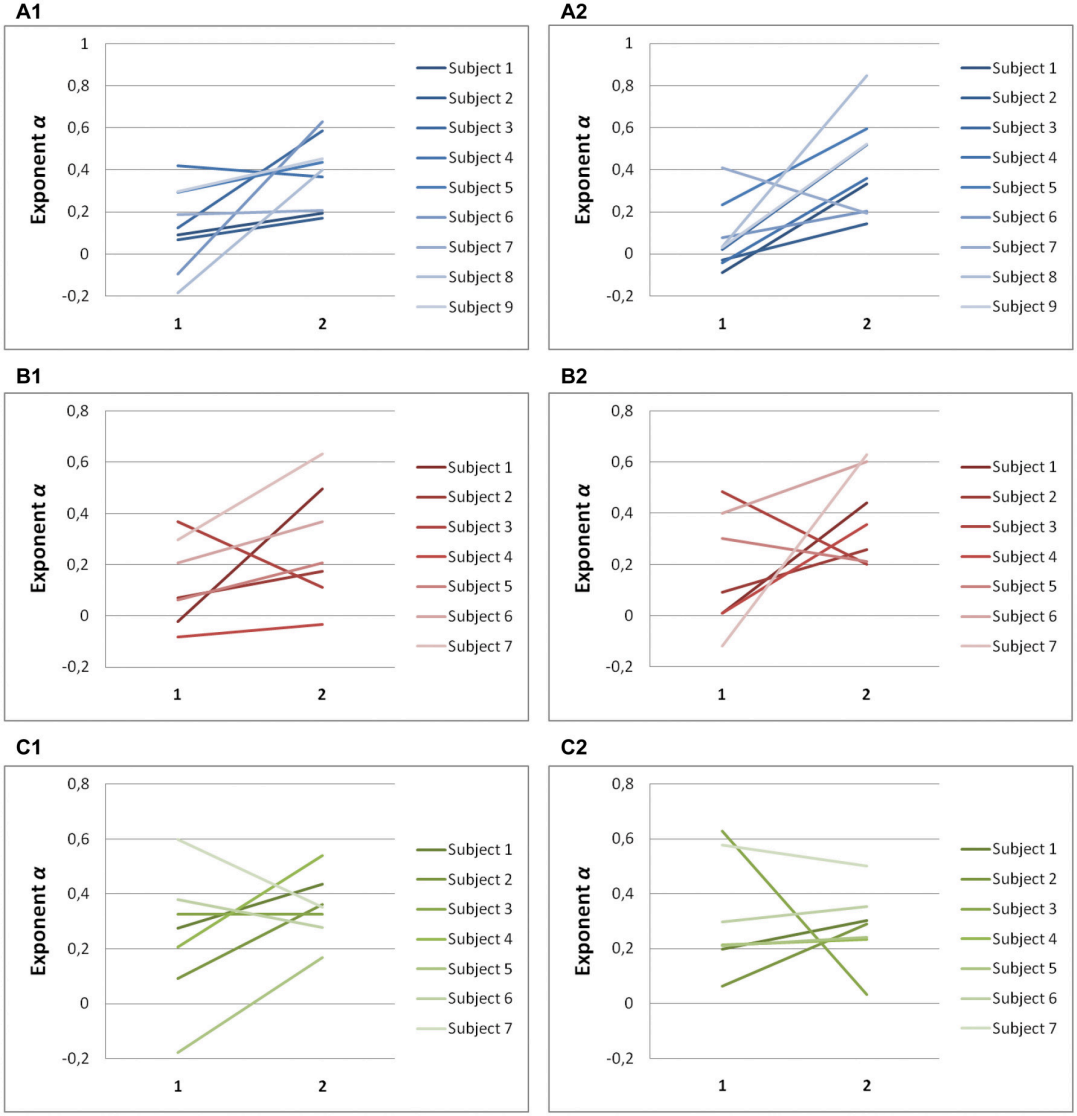
**Summary of exponent α-values of endpoint-error time series for on-axis and off-axis directions in all subjects**. The plots show the exponent α for **(A1)** rightward and **(A2)** leftward saccades to horizontal targets in Task H, **(B1)** upward and **(B2)** downward saccades to vertical targets in Task V and, **(C1)** toward ±45° and **(C2)** toward ±115° saccades to oblique targets in Task O. Exponents α of on-axis endpoint-error time series (abscissa index “1”) and off-axis endpoint-error time series (abscissa index “2”) are shown.

Significant differences in scaling exponents α are found between the two components of endpoint error (Figure 4). For predictive saccades to horizontal targets, the exponent α of the endpoint-error time series is significantly lower in the on-axis direction than in the off-axis direction (0.10 ± 0.17 vs. 0.40 ± 0.19, *P* < 0.001). For predictive saccades to vertical targets, the exponent of the endpoint-error time series is also significantly lower in the on-axis direction than in the off-axis direction (0.15 ± 0.19 vs. 0.33 ± 0.21, *P* < 0.05). For oblique targets, however, differences between on-axis and off-axis exponents were negligible (0.28 ± 0.22, 0.31 ± 0.13).

A scaling exponent of zero indicates a flat power spectrum, representative of white noise, in which successive values are uncorrelated. The slopes obtained from on-axis endpoint-error time series are not significantly different from 0 (task H, *P* > 0.05; task V, *P* > 0.05), whereas the slopes obtained from off-axis endpoint-error time series are significantly different from 0 (task H, *P* < 0.001; task V, *P* < 0.001). On the other hand, all slopes obtained from endpoint-error time series of saccades to oblique targets were significantly different from 0 (task O: *P* < 0.05).

There were no significant age effects in the scaling exponents from the H, V, and O data (first three columns in Table 1). This was the case with the exponents shown in the table as well as the paired differences between on-axis and off-axis exponents in each case. Correlation coefficients with respect to age ranged from −0.43 to 0.15, and all *P*-values were greater than 0.25.

### Hurst exponents confirm scaling results for horizontal and vertical saccades

**Figure 5:**
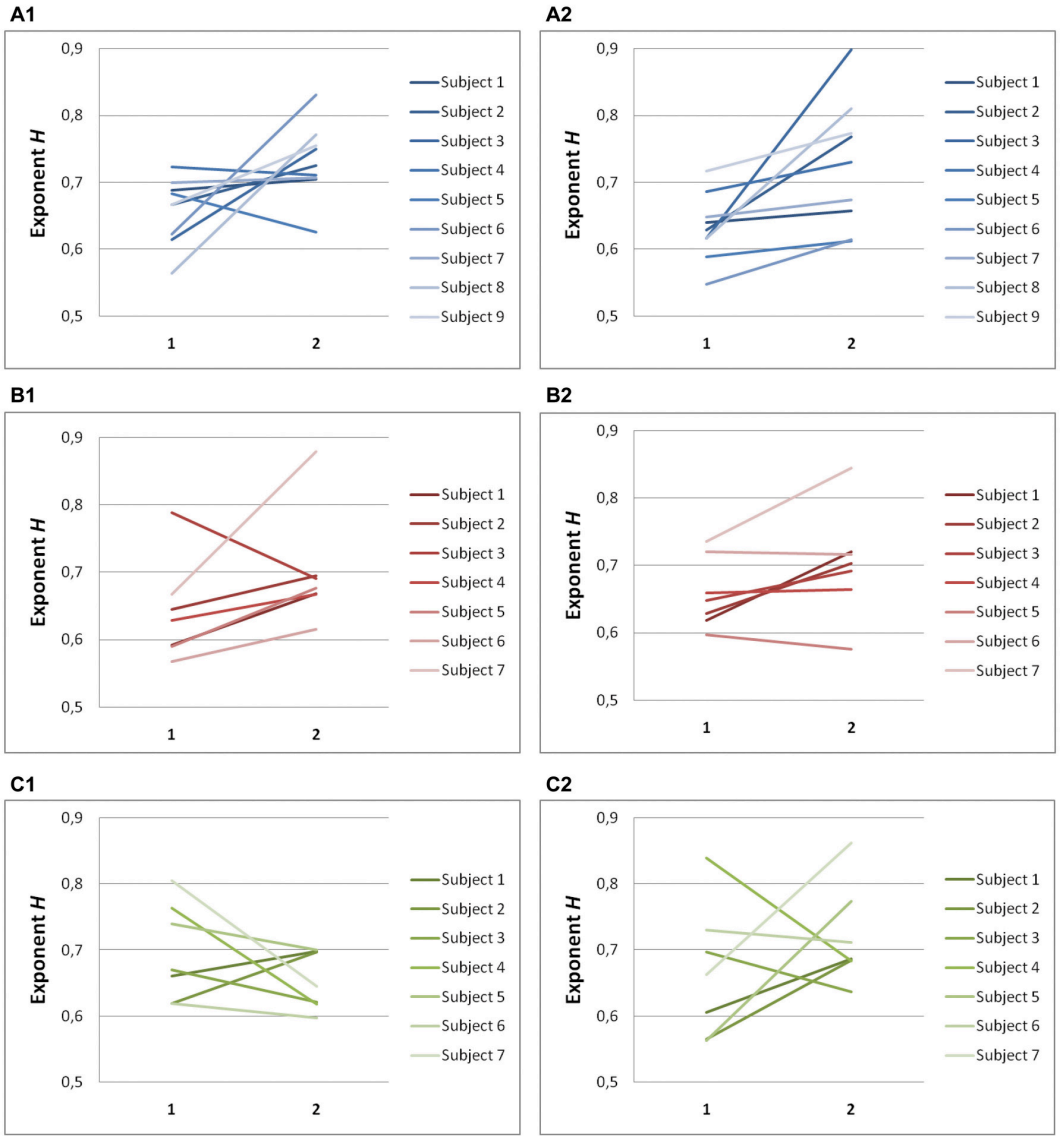
**Summary of exponent *H*-values of endpoint-error time series for on-axis and off-axis directions in all subjects**. The plots show the exponent H for **(A1)** rightward and **(A2)** leftward saccades to horizontal targets in Task *H*, **(B1)** upward and **(B2)** downward saccades to vertical targets in Task V and, **(C1)** toward ±45° and **(C2)** toward ±115° saccades to oblique targets in Task O. Exponents *H* α of on-axis endpoint-error time series (abscissa index “1”) and off-axis endpoint-error time series (abscissa index “2”) are shown.

Hurst exponents, H, showed the same trend as exponents α for each task (Table 2). In predictive saccades to horizontal and vertical targets, H was significantly lower in the on-axis direction than in the off-axis direction (task H: *P* = 0.001; task V: *P* < 0.05). In predictive saccades to oblique targets, differences in H were small and not statistically significant (Figure 5). When we compared values of H with values obtained from the expected relation between H and α, we found weak but significant agreement in the on-axis direction (*r* = 0.32, *P* = 0.03) and stronger agreement in the off-axis direction (*r* = 0.47, *P* = 0.001).

**Table 2:** **Summary of scaling Hurst (*H*) exponent values (mean and SD of *H*-values estimated for rightward and leftward saccades or upward and downward saccades in each experiment) from endpoint error in the directions along target motion (on-axis) and orthogonal to target motion (off-axis), for all subjects**.

**Subject**		**Task H Target: dot**	**Task V Target: dot**	**Task O Target: dot**	**Task LINE Target: vertical line**
		***H* on-axis/*H* off-axis**	***H* on-axis/*H* off-axis**	***H* on-axis/*H* off-axis**	***H* on-axis/*H* off-axis**
1	*H* computed	0.66 ± 0.03/0.68 ± 0.03^*^	0.61 ± 0.02/0.69 ± 0.04^*^	0.63 ± 0.04/0.69 ± 0.01^*^	0.66 ± 0.00/0.71 ± 0.06^*^
	*H* expected	0.50 ± 0.06/0.63 ± 0.05	0.50 ± 0.01/0.73 ± 0.02	0.62 ± 0.03/0.68 ± 0.05	0.53 ± 0.03/0.75 ± 0.03
2	*H* computed	0.65 ± 0.03/0.75 ± 0.03^*^	0.64 ± 0.01/0.70 ± 0.01^*^	0.59 ± 0.04/0.69 ± 0.01^*^	0.65 ± 0.04/0.82 ± 0.06^*^
	*H* expected	0.51 ± 0.03/0.58 ± 0.01	0.54 ± 0.01/0.61 ± 0.03	0.54 ± 0.01/0.66 ± 0.03	0.62 ± 0.14/0.75 ± 0.01
3	*H* computed	0.62 ± 0.00/0.82 ± 0.11^*^	0.72 ± 0.10/0.69 ± 0.00^*^	0.68 ± 0.02/0.63 ± 0.01^*^	0.64 ± 0.04/0.75 ± 0.03^*^
	*H* expected	0.54 ± 0.04/0.78 ± 0.02	0.71 ± 0.04/0.58 ± 0.03	0.74 ± 0.11/0.59 ± 0.10	0.48 ± 0.01/0.86 ± 0.07
4^a^	H computed	0.70 ± 0.03/0.72 ± 0.01^*^	0.64 ± 0.02/0.67 ± 0.00^*^	0.80 ± 0.05/0.65 ± 0.05	0.65 ± 0.03/0.79 ± 0.08^*^
	*H* expected	0.59 ± 0.16/0.68 ± 0.00	0.48 ± 0.03/0.58 ± 0.14	0.61 ± 0.00/0.69 ± 0.11	0.57 ± 0.09/0.86 ± 0.16
5^a^	H computed	0.64 ± 0.07/0.62 ± 0.01	0.59 ± 0.01/0.63 ± 0.07^*^	0.65 ± 0.13/0.74 ± 0.05^*^	0.71 ± 0.09/0.84 ± 0.01^*^
	*H* expected	0.63 ± 0.02/0.76 ± 0.06	0.59 ± 0.08/0.60 ± 0.00	0.51 ± 0.14/0.60 ± 0.03	0.66 ± 0.04/0.80 ± 0.14
6	*H* computed	0.59 ± 0.05/0.72 ± 0.15^*^	0.64 ± 0.11/0.67 ± 0.07^*^	0.67 ± 0.08/0.65 ± 0.08^*^	0.66 ± 0.05/0.78 ± 0.08^*^
	*H* expected	0.50 ± 0.06/0.71 ± 0.15	0.65 ± 0.07/0.74 ± 0.08	0.67 ± 0.03/0.66 ± 0.03	0.57 ± 0.04/0.78 ± 0.05
7	*H* computed	0.67 ± 0.04/0.69 ± 0.02	0.70 ± 0.05/0.86 ± 0.02^*^	0.73 ± 0.10/0.75 ± 0.15	0.72 ± 0.04/0.87 ± 0.09^*^
	*H* expected	0.65 ± 0.08/0.60 ± 0.01	0.54 ± 0.15/0.82 ± 0.00	0.79 ± 0.01/0.71 ± 0.05	0.70 ± 0.09/0.93 ± 0.06
8	*H* computed	0.59 ± 0.04/0.79 ± 0.03^*^			
	*H* expected	0.46 ± 0.08/0.81 ± 0.16			
9	*H* computed	0.69 ± 0.04/0.76 ± 0.01^*^			
	*H* expected	0.58 ± 0.09/0.74 ± 0.02			

Table entries are mean values of the computed exponent H, and the expected value of H obtained using the equation H = (1 + α)/2. Asterisks (^*^) indicate values of exponent H that were in line with those of exponent α. Each value in the table is the mean of the two exponents α calculated separately for either rightward and leftward saccades or for upward and downward saccades.

a *subject who skipped a saccade to one or more target jumps*.

### Line targets promote a reduction in error-control in task-irrelevant directions

Our interpretation of inter-trial scaling is that it reflects storage of error information between trials which aids in motor accuracy (see Wong and Shelhamer, 2011, and Discussion). To help refine this interpretation, we wished to provide a stimulus in which there was little incentive to correct errors in the direction orthogonal to target motion. This was done by using two vertical lines as the targets for horizontal saccades. In this way, a definite stimulus for horizontal saccade extent along the target direction (on-axis) was provided, but the stimulus for the off-axis component was indefinite. Scaling exponents α and *H* of horizontal predictive saccades were compared between the vertical-line and point targets for each subject.

**Figure 6:**
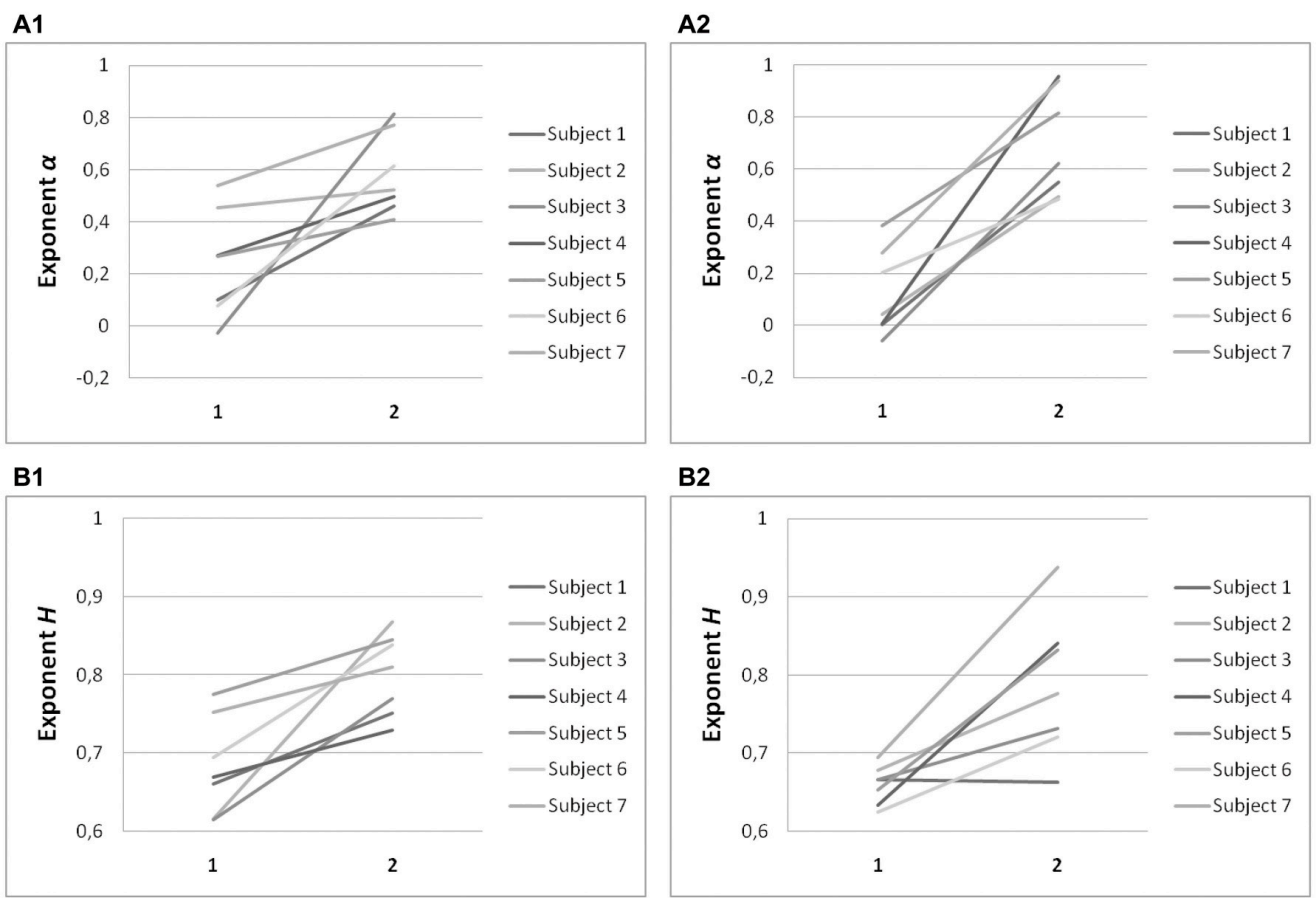
**Summary of exponent α and exponent *H*-values of endpoint-error time series for on-axis and off-axis directions in all subjects**. Plots show **(A)** exponent α and **(B)** exponent *H* of rightward **(A1, B1)** and leftward **(A2, B2)** saccades to horizontal targets in Task LINE. Exponents α and *H* of on-axis endpoint-error time series (abscissa index “1”) and off-axis endpoint-error time series (abscissa index “2”) are shown.

**Figure 7:**
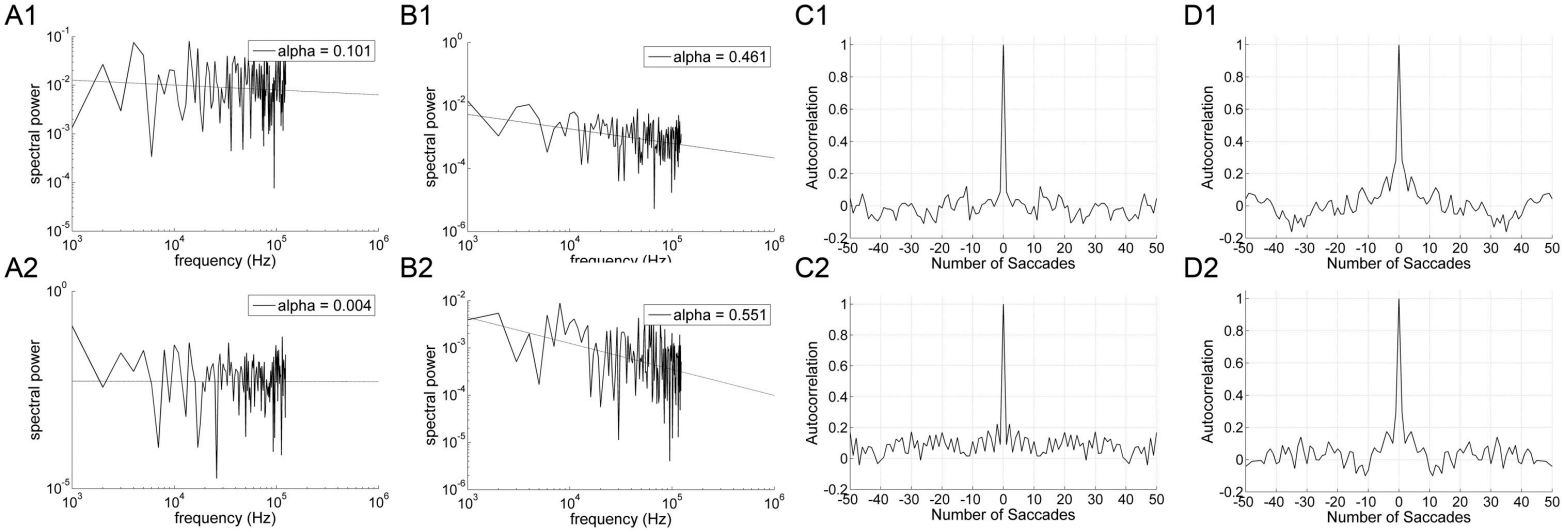
**An example of data obtained from 10° horizontal predictive saccades to a vertical line made by a representative subject**. Data from on-axis endpoint-error time series **(**columns **A** and **C)** are compared to data from off-axis endpoint-error time series **(**columns **B** and **D)**. Data from rightward (1) and leftward (2) predictive saccades are shown separately. **(A,B)** Plot of power spectra of endpoint-error time series. Power spectra are plotted on a log-log scale. The broken line shows fit to a linear polynomial equation. Values of exponent α are indicated. **(C,D)** Plot of corresponding autocorrelation functions of endpoint-error time series. Autocorrelation functions are plotted vs. the number of trials of relative shift between each endpoint-error time series and a copy of itself.

Significant differences in α (*P* < 0.001) and *H* (*P* < 0.001) were found between the on-axis and off-axis components for the vertical line, as was the case for point targets (Figure 6). There was a significantly lower value of α in the on-axis direction (0.18 ± 0.19) than in the off-axis direction (0.64 ± 0.18). The off-axis exponents were significantly different from 0 (*P* < 0.001), whereas the on-axis exponents were not (*P* > 0.05), for leftward saccades; rightward saccades showed the same trend (*P* = 0.022). The exponent *H* was also significantly lower in the on-axis direction (0.67 ± 0.05) than in the off-axis direction (0.79 ± 0.07). Figure 7 shows the power spectrum and autocorrelation function of endpoint-error time series for a subject recorded during the LINE task.

**Figure 8:**
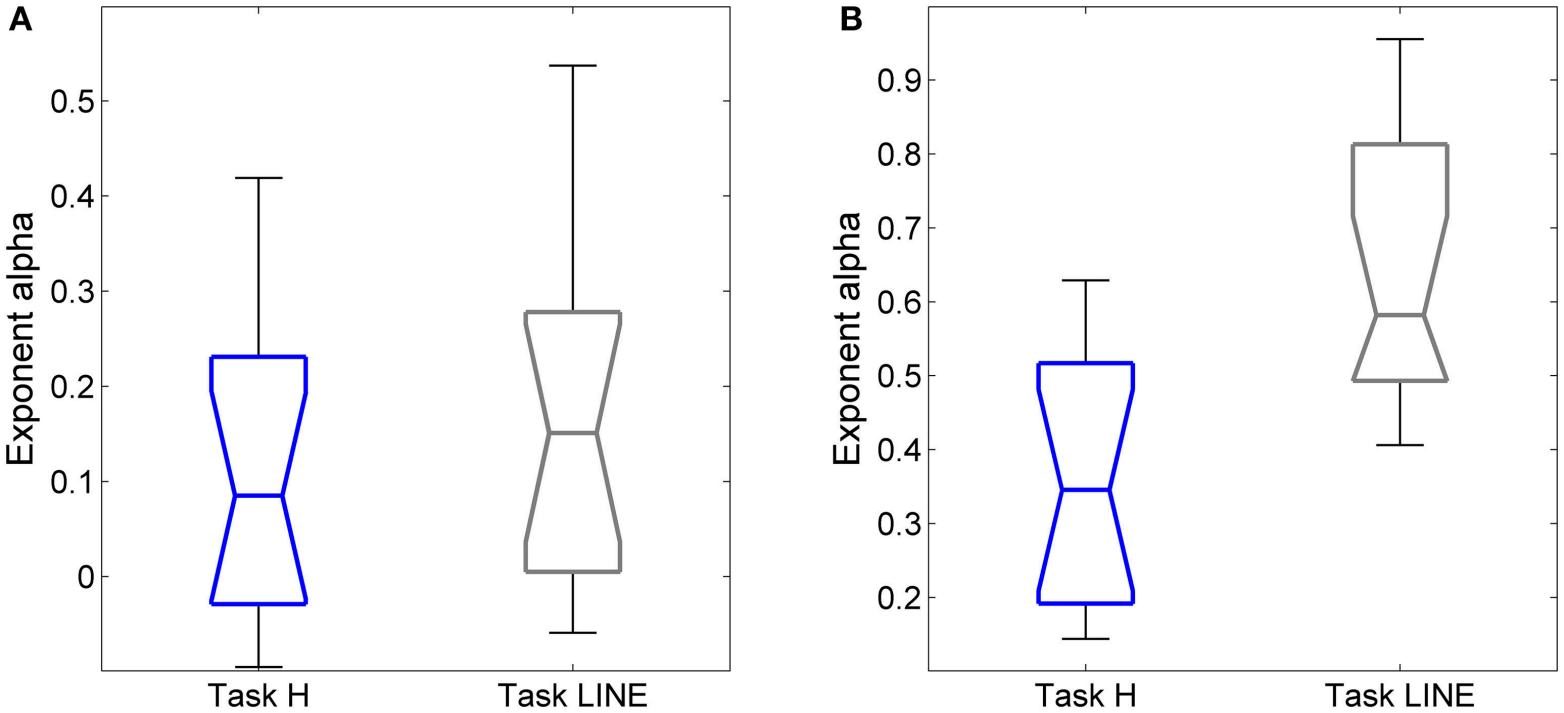
**Relationships between exponent α and target (point and vertical line in Task H and Task LINE, respectively), for horizontal predictive saccades**. Plot of exponent α values of **(A)** endpoint-error time series for on-axis and **(B)** off-axis directions in relation to task. Summary of exponent α values for each subject is shown. The box and whisker plots show exponent α of endpoint-error time series for the two targets, the point (blue) and vertical line (gray). The horizontal bar in each box represents the median and lower and upper quartile values of exponent α. The notches represent the 95% confidence interval around the median. The whiskers represent the distribution.

The exponents α for on-axis and off-axis directions were compared for the two target types (vertical line and point) for each subject (Figure 8). There was a significant difference between the off-axis exponents (point compared to line); exponents were smaller in the point task then the line task (paired *t*-test, *P* < 0.05). Differences in on-axis exponents between point and line tasks were negligible.

## Discussion

We find evidence for temporal correlations between successive endpoints in sequences of predictive saccades made to two alternating targets. More specifically, we examined these properties in sequences of saccades made in the same direction (e.g., right or left). Since we stimulated saccades with alternating horizontal targets, this required the creation of separate rightward and leftward endpoint series by extracting every other value from the total series of right/left endpoints. We did this because of known directional asymmetries in saccade control, which might confound the analyses. This process adds a separate complication, however, in that it likely modifies the inter-trial correlations in each resulting segregated endpoint series. This is because consecutive endpoints in the new series are separated by an endpoint in the opposite direction. The fact that we see consistent trends between on-axis and off-axis correlations in our data at all, despite this complication, speaks to the strength of these relationships.

For horizontal and vertical saccades, inter-trial correlations are weak or nonexistent along the direction of target motion, and strong along the direction orthogonal to target motion. The correlations that do exist are in the form of a power-law: the autocorrelation decays as τ^−β^, where τ is the lag (trial index) between trials. Likewise, the power spectrum of the endpoint series decays as a power law: *f* ^−α^, easily seen in the power spectra. This contributes to a string of findings of power-law (fractal) correlations in sequences of trials in various movement-control tasks (see Lowen and Teich, 2005 for a partial review).

Curiously, oblique saccades do not exhibit this same pattern, but instead have nearly equivalent error corrections in both the on-axis and off-axis directions. However, oblique saccades may be controlled differently than horizontal and vertical saccades, as errors in both directions are task-relevant in different ways. On-axis errors, similar to horizontal and vertical saccades, are useful for supporting the maintenance of appropriate saccade amplitude. However, the production of oblique saccades may rely more heavily on off-axis errors to produce corrections in angular direction of the saccade. Furthermore, corrections in on-axis and off-axis directions for an oblique saccade may be difficult to decouple. This is because saccade commands are typically generated by relying on separate horizontal and vertical pathways. Thus, it is difficult to identify whether an error has occurred because of an incorrect vectorized motor plan, or an inappropriate translation of the horizontal and vertical components of the motor command into a movement. Therefore, it is reasonable that on-axis inter-trial correlations for oblique saccades are not significantly smaller than those of off-axis correlations, and furthermore that it is difficult to completely extract all of the available error information to resolve oblique saccade errors. The need to coordinate two muscle pairs for oblique saccades might also contribute to differences in variability (and accuracy) between oblique movements and those in cardinal directions, although this is unlikely to be a major effect since vertical saccades are also controlled by coordinated activity between two pairs of muscles. In addition, movement errors (mean and variance) are not systematically or substantially greater for oblique saccades than for horizontal or vertical saccades. Finally, any muscle-pairing proposal is unlikely to explain the differences in inter-trial correlations between on-axis and off-axis directions, which is the primary outcome of this work.

Despite the many findings on power-law behavior in physiology, it remains an open question as to why power-law (fractal) scaling such as we see in saccadic eye movements is so prevalent. A prevailing hypothesis is that power-law correlations represent an optimal balance between stability and flexibility (Bak et al., 1988). A system that is too rigid and inflexible would not respond to incoming information, and hence might perform well under unchanging conditions but show no ability to adapt to stimulus manipulations. On the other hand, a system that is extremely flexible and adjusts immediately to each new piece of incoming information might show a great ability to rapidly adjust to stimulus manipulations, but risks losing performance stability and can end up trying to (incorrectly) adapt its behavior to environmental or measurement noise. The manner in which prior information is used to guide this trade-off between flexibility and stability is reflected in the correlations between trials. The proper balance seems to be represented in many systems by power-law correlations in trial-to-trial performance. While it is not completely clear why power-law correlations would confer such a characteristic, there is some evidence for this interpretation. In our own work (Wong and Shelhamer, 2014) we found that stronger inter-trial correlations in the amplitudes of consecutive predictive saccades (stimulated as in the present study) are associated with more rapid adjustment to target manipulations in a separate adaptation task. Thus, the stronger the power law (the less like uncorrelated white noise) in a prediction task, the more adaptable the person. Having established that power-law correlations might confer some performance advantage in consecutive amplitudes, the algebraic relationship between amplitudes and endpoints might then lead to the correlations that we see in some of our endpoint time series.

### Differences between endpoint error and amplitude error

It is important to note that there is a difference between correlations in sequences of parameter values that represent performance, and correlations in sequences that represent residuals or error. The former implies storage of performance information needed to carry out the task, by retaining information to improve subsequent trials. The latter implies the existence of unused information. It is this latter type of correlation that we examine in this study, since endpoints are primarily a representation of movement errors. There are differences in scaling between the on-axis and off-axis components of saccade endpoint errors. Furthermore, these results differ from those with saccade amplitudes, where scaling is strong in the on-axis direction of target motion. Understanding the essential distinction between amplitudes and endpoints sheds light on these disparate findings.

When the oculomotor system is presented with a visual target (or an anticipated target in the case of predictive saccades), a saccade is programmed with appropriate metrics. It is the amplitude of the saccade that is neurally controlled (e.g., Soetedjo et al., 2002), with the desired amplitude derived from the distance between the endpoint and the target. This is readily seen in experiments where the target is moved in the course of a saccade (a double-step stimulus, McLaughlin, 1967): before adaptive adjustment takes place, the saccades have large endpoint errors since they are programmed to go to the initial target position. In contrast, it is the endpoints that provide information on movement error (difference between desired and actual amplitudes). There is no visual feedback of error if there is no target to provide a basis of comparison.

Amplitudes are programmed by the oculomotor system, and predictive saccades must be programmed in advance of actual target occurrence. Therefore, information on the performance of previous saccades must be stored and processed otherwise accurate predictive saccades could not be made. Strong inter-trial correlations in consecutive amplitudes (Wong and Shelhamer, 2011) reflect this storage. Such storage is less prevalent for reactive saccades (Wong and Shelhamer, 2011). This is because information about target location is reliably presented on each trial sufficiently in advance of each saccade, instead of requiring that the subject generate a predictive movement based entirely on previous experience.

### Inter-trial correlations reflect the use of error information for controlling future movements

If greater amplitude correlations reflect better adaptation capability, how can we explain the lack of inter-trial correlations in the more-controlled on-axis direction for saccade endpoints? As noted, the endpoints of primary saccades (before any corrective saccades) reflect the actual motor-planning error in each movement. We can think of this error series as the set of residuals that is produced by an estimation process—one in which the oculomotor system is constantly estimating the next target location in order to properly program a predictive saccade to that target. If correlations exist in the residuals, then additional information is contained in that series, which could be extracted and used to improve performance. Consider a simple example of a linear regression. If there is a trend (linear relationship) that exists in the residuals, then that regression line is not the best that could have been produced. This is an established property of optimal estimators in general (Kailath, 1981): residuals should form a white-noise process. Hence, the absence of inter-trial correlations in the (residual) endpoint errors suggests that each predictive-saccade amplitude is programmed utilizing the maximum amount of available information.

We found similar results in our previous work on adaptation (Wong and Shelhamer, 2014). In that study, stronger correlations between consecutive saccade amplitudes (which represent performance, not error) were associated with better adaptation ability in a separate task. However, the residuals (errors) of the saccades that were made during the adaptation task showed the opposite association: weaker correlations were associated with better adaptation. This is because the residuals represent movement errors, and the more information that has been extracted from those errors, the weaker will be the correlation between the residuals, and the better the ability to adapt.

The fact that inter-trial correlations (scaling) do exist in the off-axis direction is equally revealing. Following similar reasoning, the presence of these correlations indicates that the estimation process is not optimal in this direction. There is information remaining in the endpoint-error series (the estimation residuals). This remaining information could have been used, presumably, to improve performance in the off-axis direction. However, since target motion is along the on-axis direction, the oculomotor system may place less emphasis on error minimization in the off-axis direction. Errors are likely to be larger in the on-axis direction and more task-relevant, and hence of greater consequence. This is confirmed by the findings with a vertical-line target, where there are even stronger inter-trial correlations in the off-axis direction. In this case even less emphasis is placed on maintaining a consistent vertical position of the eye, since any saccade that lands along the vertical extent of the line is as much “on target” as any other endpoint falling along that line.

Thus, maximal information is extracted along the direction that is of most concern, and less so along other directions. This has been found previously using different procedures (van Beers et al., 2013). In that study, subjects made reaching movements to a line, analogous to our LINE task, yielding what they describe as task-relevant (perpendicular to the line) and task-irrelevant (along the line) components. Movement errors in the task-irrelevant direction had positive lag-1 autocorrelation values, indicating at least some storage of performance from one trial to the next. (A positive lag-1 correlation means that each value is, on average, positively correlated with the subsequent value—the value at a relative delay, or lag, of one time step). In the task-relevant direction, on the other hand, the lag-1 autocorrelation was near zero, indicating uncorrelated trials, as we find also with saccade endpoint errors. This was interpreted as “effective trial-by-trial correction of motor planning on the basis of observed motor errors,” which is compatible with our interpretation of optimal estimation in generating on-axis movements. The authors interpreted the positive off-axis correlation as random effects of planning noise that accumulate over successive movements. However, if random noise is simply accumulating in the off-axis direction, it is surprising that the observed variability magnitude remains larger in the on-axis direction where errors are fully corrected. Thus, active corrections are still occurring in the off-axis direction, but are simply less rigorously maintained. By not extracting all of the available information in the estimation process on each trial to fully correct for each error, the remaining residuals accumulate and hence can appear to take the form of a random walk. Thus, our results suggest that it is not simply random noise that is accumulated, but partially corrected errors.

### Other sources of systematic variability in saccades

Signal-dependent noise is manifest as larger variability for larger movements (Harris and Wolpert, 1998). This is evident in the horizontal components of the H and V conditions, where on-axis variability is larger than off-axis variability. However, this same characteristic is also present for oblique saccades (condition O), in which the horizontal and vertical commands are nominally identical (for these 45° saccades) and thus on-axis and off-axis variability would be equal if due predominantly to signal-dependent noise. In addition, signal-dependent noise would not in itself explain the inter-trial correlation structure that we see.

Best visual acuity occurs when targets are within about half a degree of the center of the fovea. On-axis errors in this study are of this approximate size, while off-axis errors are much smaller. Therefore, one might expect that on-axis errors would be more actively controlled, given that they are flirting with the limits of highest visual acuity. This might well explain why off-axis errors, being within foveal range, are not as strongly controlled. In addition, saccades systematically fall short by about 10%. This hypometria has performance advantages (Harris and Wolpert, 2006) and thus seems to be deliberate and purposeful. This is also consistent with the greater degree of control in the on-axis direction. This intentional, consistent hypometria thus suggests that the saccadic system imposes a large degree of control in the on-axis direction.

### Study limitations

The relatively small sample size in this study (nine subjects) might limit the generalizability of the results. The small number of subjects tested is due in part to the use of the scleral search coil method to measure eye movements (see below), which can be intimidating to naive subjects. Nevertheless, consistent results were obtained in the primary outcome measures even with this small population.

Another limitation is the use of the scleral search coil. This is the method of choice when high spatial and temporal resolution and precision are required, in horizontal and vertical directions. However, the presence of the coil on the eye might alter the programming of saccadic eye movements (Frens and van der Geest, 2002). Presumably this would alter movements equally in all directions, such that the major findings here which relate mainly to differences between directions are still valid. Nevertheless, this is a confound that might be addressed in further studies.

## Conclusion

This study provides further insight regarding how movements are controlled by the central nervous system, and in particular how resources are allocated to maintain appropriate control over different aspects of movement, as needed for adequate performance. The findings support an interpretation that “optimal” control does not mean that all errors or variations are minimized over all components of motion. Rather, more care is taken in controlling movement components that are more relevant to the task at hand, and whose inaccuracy could be detrimental to the overall goals of the movements.

The study also makes a contribution to the field of fractal physiology. Findings of power-law scaling (manifest from a form of fractal time series) continue to arise in many studies of cognition and motor control. Our results find power-law scaling in task-irrelevant directions for horizontal and vertical saccades. As in so many other cases, it is not clear if this fractality is of specific importance. What is clear, however, is that there are differences in the correlation structure—and thus in the storage of error information—between different components of motion. That the stronger correlations are fractal in form matches results from our previous studies in saccade control.

## Author contributions

PF performed the experiments and data analysis, prepared a first draft of the manuscript, and participated in its editing. AW helped run experiments and perform data analysis, and contributed to the preparation of the manuscript. MS helped conceive the experiments, and supervised and contributed to the manuscript preparation.

## Funding

This work was supported by National Science Foundation Grant BCS-1126957, and National Institutes of Health Grants EY019713 and DC000023.

### Conflict of interest statement

The authors declare that the research was conducted in the absence of any commercial or financial relationships that could be construed as a potential conflict of interest.
